# Using Whole Breast Ultrasound Tomography to Improve Breast Cancer Risk Assessment: A Novel Risk Factor Based on the Quantitative Tissue Property of Sound Speed

**DOI:** 10.3390/jcm9020367

**Published:** 2020-01-29

**Authors:** Neb Duric, Mark Sak, Shaoqi Fan, Ruth M. Pfeiffer, Peter J. Littrup, Michael S. Simon, David H. Gorski, Haythem Ali, Kristen S. Purrington, Rachel F. Brem, Mark E. Sherman, Gretchen L. Gierach

**Affiliations:** 1 Barbara Ann Karmanos Cancer Institute, Detroit, MI 48201, USA; duric@karmanos.org (N.D.); plittrup@delphinusmt.com (P.J.L.); simonm@karmanos.org (M.S.S.); gorskid@med.wayne.edu (D.H.G.); purringk@karmanos.org (K.S.P.); 2 Delphinus Medical Technologies, Novi, MI 48374, USA; msak@delphinusmt.com; 3 School of Medicine, Wayne State University, Detroit, MI 48201, USA; 4 Division of Cancer Epidemiology and Genetics, National Cancer Institute, National Institutes of Health, Bethesda, MD 20892, USA; shaoqi.fan@nih.gov (S.F.); pfeiffer@mail.nih.gov (R.M.P.); 5 Henry Ford Health Systems, Detroit, MI 48202, USA; HALI1@hfhs.org; 6 Department of Radiology, George Washington University, Washington, DC 20037, USA; rbrem@mfa.gwu.edu; 7 Mayo Clinic, Jacksonville, FL 32224, USA; Sherman.Mark@mayo.edu

**Keywords:** breast neoplasms, mammographic breast density, risk factors, ultrasound tomography, sound speed

## Abstract

Mammographic percent density (MPD) is an independent risk factor for developing breast cancer, but its inclusion in clinical risk models provides only modest improvements in individualized risk prediction, and MPD is not typically assessed in younger women because of ionizing radiation concerns. Previous studies have shown that tissue sound speed, derived from whole breast ultrasound tomography (UST), a non-ionizing modality, is a potential surrogate marker of breast density, but prior to this study, sound speed has not been directly linked to breast cancer risk. To that end, we explored the relation of sound speed and MPD with breast cancer risk in a case-control study, including 61 cases with recent breast cancer diagnoses and a comparison group of 165 women, frequency matched to cases on age, race, and menopausal status, and with a recent negative mammogram and no personal history of breast cancer. Multivariable odds ratios (ORs) and 95% confidence intervals (CIs) were estimated for the relation of quartiles of MPD and sound speed with breast cancer risk adjusted for matching factors. Elevated MPD was associated with increased breast cancer risk, although the trend did not reach statistical significance (OR per quartile = 1.27, 95% CI: 0.95, 1.70; p_trend_ = 0.10). In contrast, elevated sound speed was significantly associated with breast cancer risk in a dose–response fashion (OR per quartile = 1.83, 95% CI: 1.32, 2.54; p_trend_ = 0.0003). The OR trend for sound speed was statistically significantly different from that observed for MPD (*p* = 0.005). These findings suggest that whole breast sound speed may be more strongly associated with breast cancer risk than MPD and offer future opportunities for refining the magnitude and precision of risk associations in larger, population-based studies, including women younger than usual screening ages.

## 1. Introduction

Mammographic percent density (MPD) is a strong breast cancer risk factor that typically confers a three—to fivefold elevation in risk for the highest versus lowest levels of density [1]. Given that breast cancer risk prediction models under-perform with regard to estimating individual risk, researchers have attempted to incorporate MPD into such models to improve their performance (as recently reviewed in Louro et al. [2]). Multiple studies [2] have found that adding MPD to risk models improves breast cancer risk prediction, and efforts to incorporate MPD in newer risk models are ongoing [3,4]; however, to date, improvements in discriminatory accuracy have been modest.

All current methods of MPD measurement (including tomosynthesis) are based on one or more two-dimensional projected areas of the breast rather than the full uncompressed volumes of the breast. Attempts to measure volumetric breast density (BD) in mammograms [5,6] have not substantially improved risk assessment compared to the measurement of the projected area. It is biologically implausible that the projected area of the breast should contain more information about risk than its volume, and the difficulty of trying to recover volume information from the thickness of a compressed breast likely limits the impact of MPD in breast cancer risk models. Furthermore, elevated mammographic density may produce its strongest effect among young women who are below the mammographic screening age, but who might benefit from preventive interventions [7]. Evaluating density without exposing young women to ionizing radiation is critical because of concerns that mammography induces a small but significant number of cancers [8,9]. Currently, no such approaches have been implemented in clinical practice. This is unfortunate, as increased density is known to be higher on average in younger women, and risk prediction is especially important at early ages when prevention efforts may be most influential.

A large number of studies have shown that various MRI sequences, with and without contrast, can be used to characterize BD, and comparisons with mammographic percent density (MPD) have shown a high degree of correlation [10,11,12,13,14,15,16,17]. While MRI is potentially superior to mammography for measuring BD and for use with younger women [18], it has not been adopted widely because (i) it is not used routinely for screening, meaning that a BD assessment would require a separate exam, usually with contrast, (ii) MRI continues to be expensive and therefore inaccessible to a lot of centers, and (iii) standard MRI exam times are long compared to mammography. An alternative approach that combines the benefits of radiation-free, volumetric imaging with low cost and short exam times would be highly desirable.

The potential gains in risk prediction that might be realized by using alternative measures of BD obtained through emerging non-ionizing technologies have not been fully explored. The impetus for such technologies is to advance risk stratification, and thereby improve breast cancer risk assessment and monitoring and facilitate research into the etiology and prevention of the disease. Such activities will push early detection strategies toward the long-term goal of ‘risk-based’ screening in which both the frequency and type of screening are chosen based on the level of risk [19].

Previously, we proposed a new analog to MPD based on speed of sound measurements of breast tissue derived from whole breast ultrasound tomography (UST) [1,20,21,22,23,24,25,26,27,28,29]. This method uses true volume measurements and, unlike mammography, the measure is quantitative and the method does not require exposure to ionizing radiation. In this paper, we describe the results of an exploratory case-control study aimed at assessing breast sound speed as a risk factor for breast cancer. The study builds upon our previous work showing that sound speed is a surrogate measure of BD [24,25,26,27,28,29] and, by inference, a potential risk factor for breast cancer. The motivation for our current study was to explore whether UST-derived sound speed is associated with breast cancer risk.

## 2. Materials and Methods

### 2.1. Participant Recruitment

The study population was part of a larger observational study, the Ultrasound Study of Tamoxifen, which enrolled 76 patients referred for tamoxifen treatment for clinical indications and a matched comparison group of 165 women with screen negative mammograms, aged 30–70 years, at the Barbara Ann Karmanos Cancer Institute (KCI) and Henry Ford Health Systems (HFHS) in Detroit, MI from 2011 to 2014 [24,29]. For the analysis presented herein, *n* = 15 high-risk patients referred to tamoxifen for chemoprevention were excluded, and the analysis was restricted to the baseline pre-treatment UST scans and mammograms of the unaffected breast of women with unilateral breast cancer (*n* = 61 cases) and women with no personal history of breast cancer (*n* = 165 controls).

### 2.2. Identification and Selection of Cases

Cases were identified at KCI and HFHS based on a recent diagnosis of breast cancer after routine screening. Exclusion criteria were (1) pregnant; (2) lactating; (3) with active skin infections or open chest wounds because of the open interface with the water in the imaging tank; (4) breast size more than 22 cm in diameter (limit of the size of the ring ultrasound transducer); and (5) over 350 lb of weight (weight limit, as specified by the manufacturer of the UST table). Furthermore, we excluded women who had (6) bilateral synchronous breast cancer in that a breast without radiological signs of cancer would not have been available for analysis; and (7) breast implants or reduction mammoplasty.

### 2.3. Identification and Selection of Controls

To be eligible for the control group, a screening mammogram obtained at KCI or HFHS with the recommendation to continue routine screening (i.e., Breast Imaging Reporting and Data System (BI-RADS) diagnostic score of “1” or “2”) was first identified for the woman. In addition, eligible controls had no personal history of breast cancer and had not received medications or radiation for any type of cancer, were not taking tamoxifen or raloxifene to lower breast cancer risk, and had none of the exclusion criteria given above for cases. We also excluded women who were currently taking endogenous hormones (i.e., oral contraceptives and menopausal hormone therapy), since these medications may modulate breast density. Potential controls were invited to undergo UST and were frequency matched to the case group on age, race, and menopausal status. Since we compared each participant’s mammogram to her UST scans, we attempted to schedule controls for their UST visit within approximately one month of the screen-negative mammogram date. A one-month window was justified on the basis that breast density declines with age at a rate of 1%–2% per year, which translates to <0.2% per month—well below our uncertainty of BD measurement by either mammography or UST.

At the time of the UST scan, health information was collected from cases and controls via a standard health history questionnaire administered by a research nurse, including demographics, reproductive history and menopausal status. Measured weight and height were also collected. All procedures were performed under Institutional Review Board-approved protocols at the KCI, HFHS, and National Cancer Institute, with informed consent obtained from all patients.

### 2.4. Breast Imaging

**Figure 1:**
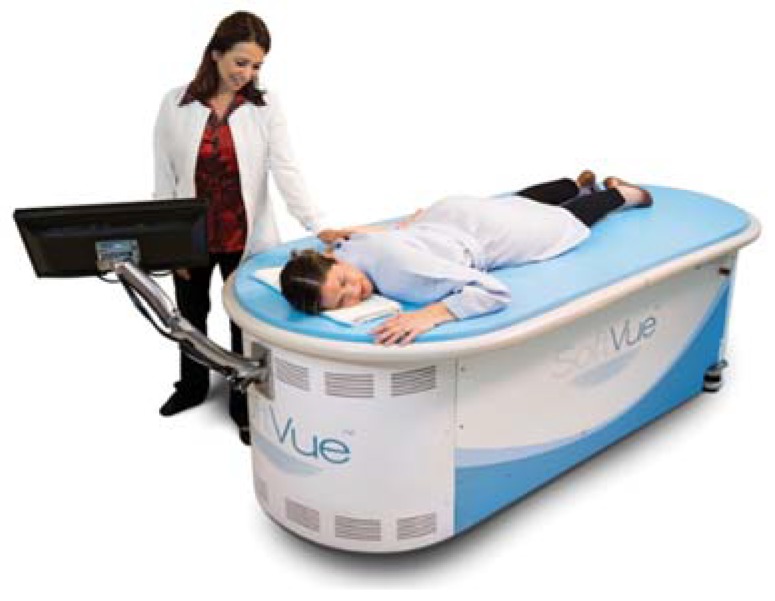
3D Whole Breast Ultrasound Tomography (UST) Scanner. UST was performed with the participant in the prone position such that the breast was suspended in a water bath containing the ultrasound sensor.

Full-field digital mammograms were obtained at KCI or HFHS. Both sites are certified by the American College of Radiology’s Mammography Accreditation Program and maintain image quality control according to the Mammography Quality Standards Act. All UST scans were performed at KCI with the SoftVue system (Figure 1), manufactured by Delphinus Medical Technologies (Novi, MI, USA) and cleared by the FDA for clinical use. As described previously [29], UST scans were performed while a patient was in the prone position with the breast to be scanned suspended in a water bath. Breasts were scanned with a 22 cm ring-shaped transducer, consisting of 2048 elements that transmit and receive ultrasound pulses. The transducer was mounted on an automated gantry that progressively captured 40–100 coronal image slices beginning at the chest wall and progressing to the nipple. Unlike conventional ultrasound, UST provides four tomographic images: reflection, enhanced reflection, stiffness, and sound speed images. The present analysis focused on volumetric breast density as estimated through sound speed images as described in further detail below.

### 2.5. Breast Density Assessment

In cases, BD was measured pre-treatment in the contralateral breast to avoid the potential influences of tumor-related changes on MPD or sound speed. For controls, we randomly selected a breast for both mammographic and UST assessment, since concurrent mammographic density measurements of left and right breasts from the same individuals have been reported to be highly correlated and predictive of risk irrespective of side (laterality) [30]. Observers were “masked” to case-control status for mammographic and UST sound speed measurements.

### 2.6. Mammography-Defined BD Measures

Craniocaudal (CC) mammographic views were analyzed using Cumulus [31], a computer-assisted thresholding software package, to generate quantitative estimates of absolute dense area (cm^2^) and total breast area (cm^2^). MPD was calculated as the dense area divided by the total breast area multiplied by 100. The area-based MPD measures estimated from Cumulus have been strongly and consistently associated with elevated breast cancer risk in numerous epidemiologic studies [1,32]. A single CC view was selected as prior studies of women evaluating both pre-diagnostic CC and MLO views in relation to risk have demonstrated that MPD is a general marker of risk that is not specific to mammographic view [30]. A reevaluation of 33 randomly selected mammograms demonstrated moderate to good reliability, yielding intraclass correlation coefficients (ICC) of 69% for dense area, 97% for total breast area, and 77% for percent MPD.

### 2.7. UST Imaging of Sound Speed

**Figure 2:**
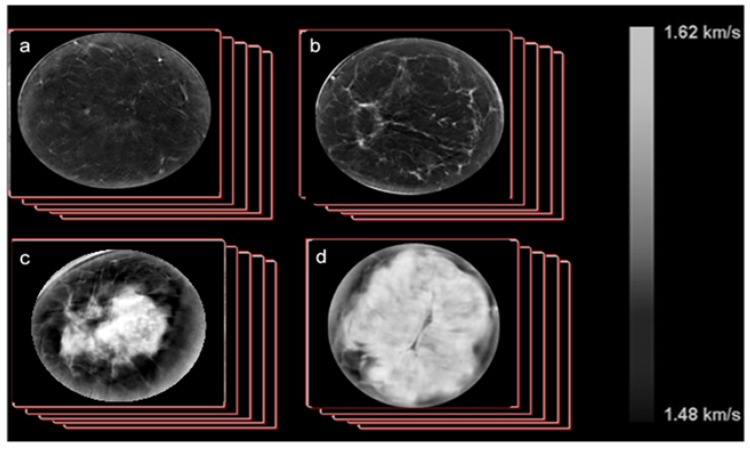
Example SoftVue image stacks of sound speed, as shown for cases ranging across the four Breast Imaging Reporting and Data System (BI-RADS) breast density categories ((**a**), fatty; (**b**), scattered; (**c**), heterogeneously dense; (**d**), extremely dense). Note the quantitative scale indicating that absolute measurements are obtained.

Data were reconstructed from the raw data collected by UST and output as DICOM images which were viewed on a standard display workstation (Figure 2). The volume averaged sound speed of the breast (VASS) was calculated using techniques previously developed [1,20,21,22,23,24,25,26,27,28,29] and summarized as follows:

- Calculate the volume of the breast, V, through a direct pixel count using previously developed automated scripts.
- Calculate the volume averaged sound speed (VASS) for each stack by summing up all the pixel values and dividing by the volume determined above using our automated script.
- Apply this calculation to image stacks (approximately from 40 to 100 coronal slices per scan) from all cases and controls.

A previous reproducibility study in a subset of 22 participants demonstrated that UST sound speed estimates were highly reliable (ICC = 93.4%) [29].

### 2.8. Statistical Analysis

Percentiles of all variables were defined based on their distribution among controls. Associations between participant characteristics and quartiles of MPD or VASS were estimated among controls using chi-square or Fisher’s exact tests for categorical variables and the Kruskal–Wallis test for continuous variables. Odds ratios (ORs) and 95% confidence intervals (CIs) for the association between quartiles of MPD and VASS with breast cancer risk were estimated using logistic regression, adjusting for matching factors (age in categories, as an ordinal trend (<45, 45–<50, 50–<55, ≥55 years), race (white, black, other), menopausal status (premenopausal, postmenopausal). Participant characteristics were considered as potential confounding factors in multivariable logistic regression models. In sensitivity analyses, we adjusted models mutually for MPD and VASS. Although body mass index (BMI, kg/m^2^) did not differ by case-control status, in sensitivity analyses, we additionally adjusted for BMI given its strong inverse association with BD.

We compared the strength of association between MPD and VASS with breast cancer risk using a bootstrap approach as follows. First, we resampled the same numbers of cases and controls as in the original dataset randomly with replacement. We then fit two logistic regression models, one with quartiles of MPD and the other one with quartiles of VASS as the exposure and estimated the respective log ORs. This was repeated 1000 times. Then, we computed the square of the difference of the log ORs based on the original dataset and divided that difference by the sum of the variances of the log ORs minus two times their covariance. The estimate of the variance covariance matrix of the estimates was the empirical bootstrap variance covariance matrix. We compared that ratio to a Chi-square distribution with one degree of freedom to obtain a *p*-value for differences in the ORs. Analyses were performed using SAS V9.4 (SAS, Cary, NC, USA); statistical tests were two-sided, and *p*-values < 0.05 were considered statistically significant.

## 3. Results

### 3.1. Patient Characteristics

The epidemiological attributes of the cases and controls are summarized in Supplementary Table S1. The median (range) age at diagnosis was 50.6 (30.2, 69.1) and the matched controls had a similar age distribution; 70% of cases and 56% of controls were premenopausal. The majority of cases (56%) and controls (66%) were black, and nearly half of cases (49%) and controls (50%) were obese (i.e., BMI 30+ kg/m^2^). Median (range) time between the mammogram and UST scan was 4.1 (0.5, 13.0) months for cases and 1.2 (0.2, 4.3) months for controls.

The relationship of participant characteristics among controls with quartiles of VASS and MPD are shown in Table 1 and Table 2. Both VASS and MPD were statistically significantly inversely associated with age, BMI, and menopause. As expected, quartiles of both VASS and MPD were strongly and positively associated with BI-RADS density categories (*p* < 0.0001), as well as with each other (*p* < 0.0001). As we have previously shown among these controls, continuous VASS measures were also strongly and positively correlated with MPD (*r* = 0.72, *p* < 0.001) [24].

### 3.2. Relation between Breast Density and Breast Cancer Risk

The distributions of MPD and VASS, categorized in quartiles determined based upon the control distribution, by case-control status are shown in Table 3.

**Figure 3:**
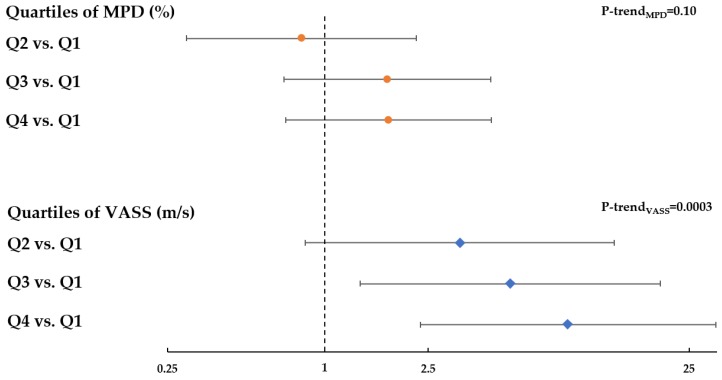
Adjusted odds ratios (ORs) and 95% confidence intervals (CIs) for the relation of quartiles of MPD (orange circle) and UST volume-averaged sound speed (blue diamond) with breast cancer risk. Quartiles were defined based upon distribution among controls. OR and 95% CI were estimated from logistic regression model adjusted for age, race, and menopausal status.

In multivariable models adjusting for matching factors, increased MPD was associated with elevated breast cancer risk compared to controls, consistent with previous studies, although the trend did not reach statistical significance (OR per quartile = 1.27, 95% CI: 0.95, 1.70; *p*_trend_ = 0.10) (Figure 3). In contrast, elevated sound speed was significantly associated with increased breast cancer risk in a dose–response fashion (OR per quartile = 1.83, 95% CI: 1.32, 2.54; *p*_trend_ = 0.0003) (Figure 3). Findings from sensitivity analyses additionally adjusting for BMI were consistent with those models adjusting only for matching factors, such that with additional adjustment for BMI, the OR per quartile of MPD was 1.34 (95% CI: 0.98, 1.84); *p*_trend_ = 0.07, and the OR per quartile of VASS was 2.06 (95% CI: 1.43, 2.95); *p*_trend_ < 0.0001 (Supplementary Table S2).

Furthermore, using a bootstrap approach, we determined that the OR trend for sound speed shown in Figure 3 was statistically significantly different from that observed for MPD (*p* = 0.005). With mutual adjustment, the risk association for MPD was null, whereas the risk association for SS remained statistically significantly elevated, both with and without adjustment for BMI (Supplementary Table S2).

## 4. Discussion

The goal of this study was to explore VASS as a potential new UST-based risk factor for breast cancer. Herein, we observed that while both elevated MPD and UST breast sound speed were associated with increased breast cancer risk, the magnitude of the risk association was stronger for UST as compared with MPD in this case-control study. Having dense breasts is very common [33]; thus, even small improvements in the accuracy of risk assessment may translate into a significant impact on the utility of sound speed for risk stratification at the population level.

The lack of statistical significance for the OR trend for MPD is not necessarily surprising given that BMI in our study population tended to be elevated and the BD distribution skewed toward lower breast densities. Based on a post hoc power calculation, we had 80% power to detect an OR associated with elevated MPD of 3.2 or higher comparing the highest to the lowest MPD quartile, for the given sample sizes of 61 cases and 165 controls. While the sample size, demographics, and skewed density distribution limit the generalizability of these findings to the larger US population, the assessment of the performance of VASS relative to MPD is valid for this particular population. The fact that strong ORs are obtained for VASS, despite operating within a narrow BD range skewed toward lower breast densities, suggests that a larger magnitude of association associated with elevated VASS may be observed in the general population of women, where dense breasts are more prevalent. Furthermore, although our breast cancer cases were patients referred to tamoxifen for clinical indications, all breast imaging was obtained prior to treatment initiation, and prior research suggests that elevated MPD is related to increased risk of both ER+ and ER− breast cancers [34], giving broad relevance to BD as a general marker of risk. To fully assess the potential of VASS as a risk factor for improving risk model accuracy, a larger, population-based study will be required to refine the magnitude as well as to increase the precision of the risk estimates.

Our results suggest that VASS has a stronger dependence on breast cancer risk than MPD and therefore has the potential to increase precision in standard risk models. Current methods of measuring BD based on mammography, either by radiologist’s estimation or computer-assisted measurement, limit the risk stratification achievable by the inclusion of BD in risk models [2]. Potential reasons for the stronger observed effects for VASS versus MPD include the true volumetric nature of BD assessment by UST of the uncompressed breast. Indeed, prior work has shown that VASS is strongly correlated with other volumetric breast imaging metrics, such as automated volumetric measures from mammography [28] as well as MRI percent water content [25]. However, in contrast to MRI, UST is a cost-effective (similar price as digital mammography) and rapid imaging strategy, requiring a water source and computer to complete the scan in 2–3 min (versus up to 45 min for MRI), without contrast or a specialized exam room. In addition, sound speed within the breast is an objective measurement that likely reflects the biophysical properties of the breast tissue and is fixed to an external standard, and is thereby relatively unaffected by day-to-day performance factors [29]. Prior studies simultaneously comparing mammographic density assessment methods have demonstrated MPD measured by Cumulus as well as by automated and visual density assessment methods to be valid methods with respect to evaluating breast cancer risk [35,36]. Future work comparing risk associations for UST sound speed to more contemporary automated approaches for density assessment on digital mammography will be important. More refined risk models will lead to greater risk stratification, which may ultimately lead to improved strategies for personalized breast cancer screening [19]. Motivations include identifying women at (i) extremely high risk, who are potential candidates for risk-reducing treatment or preventive therapy, (ii) moderately enhanced risk who might benefit from supplemental screening, and (iii) sufficiently low risk to warrant less frequent screening. Furthermore, risk stratification could be expanded to younger women where UST assessments would enable risk-based screening without ionizing radiation concerns. In the USA, 70 million women between the ages of 18 and 40 fall into this category.

The results of this study have several important implications. First, findings will inform future longitudinal studies aimed at establishing temporal relationships between UST breast sound speed and breast cancer risk, ultimately facilitating the incorporation of validated UST-derived parameters into evolving risk models. Genome-wide association studies in young women are in progress to define the genetic susceptibility loci associated with BD and breast cancer risk, and UST may allow the further characterization of this phenotype in larger numbers of young women at relatively low cost. Research to examine the effects of potential preventive interventions for breast cancer would also become possible at earlier ages and at multiple times points with UST [37], since there is no radiation exposure, and with less measurement error, than is possible with mammography. In addition, the development of CADx tools with radiomics features (e.g., pattern and texture analytics) may allow the exploration of (i) more complex relationships between UST parameters and breast cancer risk, and (ii) parameters based on parenchymal patterns to further strengthen associations with breast cancer risk. Future studies of UST-based risk factors will also have the potential to dovetail with the FDA’s increasing recognition and approval of imaging biomarkers as endpoints in clinical trials [38]. Trials are ongoing to assess the role of UST as an adjunctive screening tool among women with dense breasts.

In conclusion, this exploratory case-control study showed that increasing quartiles of whole breast volume averaged sound speed were consistently and more strongly associated with increasing breast cancer risk than quartiles of mammographic percent density. These findings suggest future opportunities for refining the magnitude and precision of UST–breast cancer risk associations, using a non-ionizing imaging modality, in larger population-based studies.

## Figures and Tables

**Table 1:** Relation of participant characteristics with ultrasound tomography-derived volume averaged sound speed (VASS) among controls (*n* = 165).

	Volume Averaged Sound Speed (VASS)								
	Quartile 1: <1440.63		Quartile 2: 1440.63 to <1445.65		Quartile 3: 1445.65 to <1452.81		Quartile 4: ≥1452.81		
Participant Characteristics	*N*	%	*N*	%	*N*	%	*N*	%	*p*-Value *
Age: Median (range)	54.2	(32.7, 67)	50.9	(40.7, 67.7)	49.6	(35.4, 69.2)	48.4	(36.8, 64.8)	0.03 **
Race									
White	12	30.0	11	25.6	12	29.3	16	39.0	
Black	27	67.5	30	69.8	28	68.3	24	58.5	0.91 ^†^
Other	1	2.5	2	4.7	1	2.4	1	2.4	
BMI, kg/m^2^									
<25	3	7.5	6	14.3	6	14.6	21	51.2	<0.0001
25–30	10	25.0	12	28.6	12	29.3	12	29.3	
30+	27	67.5	24	57.1	23	56.1	8	19.5	
Education									
At most, high school/GED	14	35.0	14	32.6	7	17.1	11	26.8	0.54
Some college/postsecondary courses	11	27.5	16	37.2	18	43.9	14	34.2	
College/graduate school	15	37.5	13	30.2	16	39.0	16	39.0	
Age at menarche									
≤12	26	66.7	27	62.8	22	53.7	19	46.3	0.34
13	9	23.1	6	14.0	11	26.8	12	29.3	
14+	4	10.3	10	23.3	8	19.5	10	24.4	
Age at first birth									
Nulliparous/≥30	13	32.5	14	32.6	12	29.3	15	36.5	0.92
<30	27	67.5	29	67.4	29	70.7	26	63.4	
Menopausal status									
Premenopausal	14	35.0	22	51.2	26	63.4	31	75.6	0.0019
Postmenopausal	26	65.0	21	48.8	15	36.6	10	24.4	
Any first degree relative with breast cancer									
No	30	75.0	32	74.4	39	95.1	32	78.1	0.056
Yes	10	25.0	11	25.6	2	4.9	9	22.0	
BI-RADS breast density									
a (entirely fat)	20	50.0	14	32.6	6	14.6	1	2.4	<0.0001
b (scattered densities)	19	47.5	27	62.8	26	63.4	7	17.1	
c (heterogeneously dense)	1	2.5	2	4.7	8	19.5	26	63.4	
d (extremely dense)	0	0.0	0	0.0	1	2.4	7	17.1	
Mammographic percent density, quartiles									
<7.8%	17	42.5	18	41.9	6	14.6	0	0.0	<0.0001
7.8 to <16.9%	16	40.0	12	27.9	13	31.7	1	2.4	
16.9 to <30.8%	7	17.5	8	18.6	15	36.6	10	24.4	
≥30.8%	0	0.0	5	11.6	7	17.1	30	73.2	

Quartiles based upon distribution among controls; * *p*-values from Chi-square test except where noted; ** Kruskal–Wallis test; ^†^ Fisher’s exact test. BI-RADS: Breast Imaging Reporting and Data System.

**Table 2:** Relation of participant characteristics with mammographic percent density (MPD) in quartiles among controls (*n* = 165).

	Quartile 1: <7.8%		Quartile 2: 7.8% to <16.9%		Quartile 3: 16.9% to <30.8%		Quartile 4: ≥30.8%		
Participant Characteristics	*N*	%	*N*	%	*N*	%	*N*	%	*p*-Value *
Age: Median (range)	53.3	(32.7, 68.5)	53.6	(35.4, 69.1)	50.4	(30.2,70.8)	48.5	(30.5, 64.8)	0.018 **
Race									
White	11	26.8	11	26.2	14	35.0	15	35.7	0.93 ^†^
Black	28	68.3	30	71.4	25	62.5	26	61.9	
Other	2	4.9	1	2.4	1	2.5	1	2.4	
BMI, kg/m^2^									
<25	2	5.0	4	9.5	10	25.0	20	47.6	<0.0001
25–30	9	22.5	11	26.2	15	37.5	11	26.2	
30+	29	72.5	27	64.3	15	37.5	11	26.2	
Education									
At most, high school/GED	12	29.3	15	35.7	10	25.0	9	21.4	0.86
Some college/postsecondary courses	15	36.6	13	31.0	14	35.0	17	40.5	
College/graduate school	14	34.2	14	33.3	16	40.0	16	38.1	
Age at menarche									
≤12	30	75.0	21	50.0	21	52.5	22	52.4	0.14
13	6	15.0	14	33.3	9	22.5	9	21.4	
14+	4	10.0	7	16.7	10	25.0	11	26.2	
Age at first birth									
Nulliparous/≥30	14	34.2	15	35.7	11	27.5	14	33.3	0.87
<30	27	65.9	27	64.3	29	72.5	28	66.7	
Menopausal status									
Premenopausal	17	41.5	21	50.0	26	65.0	29	69.1	0.039
Postmenopausal	24	58.5	21	50.0	14	35.0	13	31.0	
Any first degree relative with breast cancer									
No	32	78.1	35	83.3	35	87.5	31	73.8	0.42
Yes	9	22.0	7	16.7	5	12.5	11	26.2	
BI-RADS breast density									
a (entirely fat)	26	63.4	11	26.2	3	7.5	1	2.4	<0.0001
b (scattered densities)	15	36.6	28	66.7	21	52.5	15	35.7	
c (heterogeneously dense)	0	0.0	2	4.8	15	37.5	20	47.6	
d (extremely dense)	0	0.0	1	2.4	1	2.5	6	14.3	
Quartiles of baseline sound speed (m/s)									
<1440.63	17	41.5	16	38.1	7	17.5	0	0.0	<0.0001
1440.63 to <1445.65	18	43.9	12	28.6	8	20.0	5	11.9	
1445.65 to <1452.81	6	14.6	13	30.9	15	37.5	7	16.7	
≥1452.81	0	0.0	1	2.4	10	25.0	30	71.4	

Quartiles based upon distribution among controls; * *p*-values from Chi-square test except where noted; ** Kruskal–Wallis test; ^†^ Fisher’s exact test.

**Table 3:** Quartiles * of mammographic percent density and UST sound speed by case-control status.

	Case		Control	
	(*N* = 61)		(*N* = 165)	
	*N*	%	*N*	%
Quartiles * of MPD, %				
<7.8	10	16.4	41	24.9
7.8 to <16.9	9	14.8	42	25.5
16.9 to <30.8	20	32.8	40	24.2
≥30.8	22	36.1	42	25.5
Quartiles * of VASS, m/s				
<1440.6	3	4.9	40	24.2
1440.6 to <1445.6	11	18	43	26.1
1445.6 to <1452.8	17	27.9	41	24.9
≥1452.8	30	49.2	41	24.9

* Quartiles were defined based upon distribution among controls. CI, confidence interval; MPD, mammographic percent density; OR, odds ratio; VASS, volume averaged sound speed.

## Supplementary Materials

The following are available online at https://www.mdpi.com/2077-0383/9/2/367/s1, Table S1: Distribution of risk factors by case-control status among women undergoing ultrasound tomography, Detroit, MI. Table S2. Multivariable odds ratios (OR) and 95% confidence intervals (CIs) for the relation of MPD and VASS with breast cancer risk with and without mutual adjustment.
