# Supplementary material for: Using Whole Breast Ultrasound Tomography to Improve Breast Cancer Risk Assessment: A Novel Risk Factor Based on the Quantitative Tissue Property of Sound Speed
Source: J Clin Med. 2020 Jan 29;9(2):367. doi: 10.3390/jcm9020367 (PMC7074100; doi:10.3390/jcm9020367)
Supplement: Supplementary file 1 [file jcm-09-00367-s001.pdf]

Supplementary Table S1. Distribution of risk factors by case-control status among women undergoing ultrasound tomography, Detroit, MI.

	**Case (*N* = 61)**		**Comparison (*N* = 165)**		
	N	**%**	***N***	**%**	***p*-Value ***
Age, years					
≤50	29	47.5	77	46.7	0.91
>50	32	52.5	88	53.3	
Median (Range)	50.6 (30.2, 69.1)		51.1 (32.7, 69.2)		0.66 †
Race					
White	21	34.4	51	30.9	0.08
Black	34	55.7	109	66.1	
Other	6	9.8	5	3.0	
BMI, kg/m^2					
<25	15	24.6	36	22.0	0.91
25–30	16	26.2	46	28.1	
30+	30	49.2	82	50.0	
Education					
At most, high school/GED	19	31.2	46	27.9	0.68
Some college/postsecondary courses	18	29.5	59	35.8	
College/graduate school	24	39.3	60	36.4	
Age at menarche					
≤12	33	54.1	94	57.3	0.36
13	11	18.0	38	23.2	
14+	17	27.9	32	19.5	
Age at first birth					
Nulliparous/≥30	20	32.8	54	32.73	0.99
<30	41	67.2	111	67.27	
Menopausal status					
Premenopausal	43	70.5	93	56.4	0.054
Postmenopausal	18	29.5	72	43.6	
Any first degree relative with breast cancer					
No	45	73.8	133	80.6	0.26
Yes	16	26.2	32	19.4	
Time between baseline mammogram and baseline sound speed scan					
Month, median (Range)	4.1 (0.5, 13.0)		1.2 (0.2, 4.3)		<0.0001 †

BMI: body mass index. * *p*-values from Chi-square test except where noted; † Wilcoxon test. $n = 1$ control was missing BMI

Supplementary Table S2. Multivariable odds ratios (OR) and 95% confidence intervals (CIs) for the relation of MPD and VASS with breast cancer risk with and without mutual adjustment.

	Separate Risk Associations for MPD and VASS			Mutually Adjusted Risk Associations (MPD and VASS in the Same Model)		
	**OR**	**95% CI**	p_{trend}	**OR**	**95% CI**	p_{trend}
**Models Adjusting for Matching Factors §:**						
Quartiles * of MPD, %						
<7.8	Ref			Ref		
7.8 to <16.9	0.82	(0.30, 2.26)		0.67	(0.23, 1.96)	
16.9 to <30.8	1.75	(0.70, 4.35)		0.82	(0.28, 2.40)	
≥30.8	1.76	(0.71, 4.35)		0.51	(0.15, 1.74)	
TREND (per quartile)	1.27	(0.95, 1.70)	0.10	0.80	(0.54, 1.18)	0.26
Quartiles * of VASS, m/s						
<1440.6	Ref			Ref		
1440.6 to <1445.6	3.31	(0.85, 12.92)		3.33	(0.85, 13.12)	
1445.6 to <1452.8	5.16	(1.37, 19.40)		5.77	(1.46, 22.77)	
≥1452.8	8.60	(2.33, 31.72)		12.00	(2.64, 54.52)	
TREND (per quartile)	1.83	(1.32, 2.54)	0.0003	2.16	(1.38, 3.35)	0.0006
**Models with additional adjustment for BMI ¥:**						
Quartiles * of MPD, %						
<7.8	Ref			Ref		
7.8 to <16.9	0.83	(0.30, 2.31)		0.66	(0.22, 1.94)	
16.9 to <30.8	1.90	(0.74, 4.88)		0.89	(0.29, 2.64)	
≥30.8	2.04	(0.77, 5.42)		0.59	(0.17, 2.03)	
TREND (per quartile)	1.34	(0.98, 1.84)	0.07	0.84	(0.56, 1.26)	0.40
Quartiles * of VASS, m/s						
<1440.6	Ref			Ref		
1440.6 to <1445.6	3.65	(0.92, 14.48)		3.69	(0.93, 14.75)	
1445.6 to <1452.8	5.75	(1.50, 21.96)		6.27	(1.55, 25.35)	
≥1452.8	12.08	(3.03, 48.18)		15.20	(3.17, 72.84)	
TREND (per quartile)	2.06	(1.43, 2.96)	<0.0001	2.31	(1.46, 3.65)	0.0004

Quartiles were defined based upon distribution among controls. BMI, body mass index (kg/m^2); CI, confidence interval; MPD, mammographic percent density; OR, odds ratio; VASS, volume averaged sound speed. § OR and 95% CI were estimated from logistic regression model adjusted for age, race, and menopausal status. ¥ OR and 95% CI were estimated from logistic regression model adjusted for age, race, menopausal status and BMI; *n* = 1 control was missing BMI.
